# Single-cell analysis of adult human heart across healthy and cardiovascular disease patients reveals the cellular landscape underlying SARS-CoV-2 invasion of myocardial tissue through ACE2

**DOI:** 10.1186/s12967-023-04224-1

**Published:** 2023-05-31

**Authors:** Cong Chen, Jie Wang, Yong-Mei Liu, Jun Hu

**Affiliations:** Guang’anmen Hospital, China Academy of Chinese Medicine Sciences, Beijing, 100053 China

**Keywords:** COVID-19, SARS-CoV-2, ACE2, ADAM17, CTSL, Heart, scRNA-seq

## Abstract

**Background:**

The distribution of ACE2 and accessory proteases (ANAD17 and CTSL) in cardiovascular tissue and the host cell receptor binding of severe acute respiratory syndrome coronavirus 2 (SARS-CoV-2) are crucial to understanding the virus’s cell invasion, which may play a significant role in determining the viral tropism and its clinical manifestations.

**Methods:**

We conducted a comprehensive analysis of the cell type-specific expression of ACE2, ADAM17, and CTSL in myocardial tissue from 10 patients using RNA sequencing. Our study included a meta-analysis of 2 heart single-cell RNA-sequencing studies with a total of 90,024 cells from 250 heart samples of 10 individuals. We used co-expression analysis to locate specific cell types that SARS-CoV-2 may invade.

**Results:**

Our results revealed cell-type specific associations between male gender and the expression levels of ACE2, ADAM17, and CTSL, including pericytes and fibroblasts. AGT, CALM3, PCSK5, NRP1, and LMAN were identified as potential accessory proteases that might facilitate viral invasion. Enrichment analysis highlighted the extracellular matrix interaction pathway, adherent plaque pathway, vascular smooth muscle contraction inflammatory response, and oxidative stress as potential immune pathways involved in viral infection, providing potential molecular targets for therapeutic intervention. We also found specific high expression of IFITM3 and AGT in pericytes and differences in the IFN-II signaling pathway and PAR signaling pathway in fibroblasts from different cardiovascular comorbidities.

**Conclusions:**

Our data indicated possible high-risk groups for COVID-19 and provided emerging avenues for future investigations of its pathogenesis.

***Trial registration*:**

(Not applicable).

**Supplementary Information:**

The online version contains supplementary material available at 10.1186/s12967-023-04224-1.

## Introduction

As the coronavirus disease 2019 (COVID-19) pandemic enters its third year, the disease’s impact on the cardiovascular system is becoming increasingly evident [1]. COVID-19 is caused by SARS-CoV-2 infection, and angiotensin-converting enzyme 2 (ACE2) is the key receptor for this virus to enter cells. In addition to mediating the interaction between host cells and the SARS-CoV-2 spike (S) glycoprotein, ACE2 has a homeostatic function in regulating the renin-angiotensin-aldosterone system (RAAS), which is pivotal for both the cardiovascular and immune systems [2, 3]. ACE2 expression has been reported to correlate with increased viral load in human cell lines and mice [4]. Therefore, ACE2 may be a key link between SARS-CoV-2 infection and cardiovascular disease (CVD) [5, 6, 7, 8].

Nasal and pulmonary epithelial cells are thought to be the first cells to be infected by SARS-CoV-2, after initial viral replication and circulation, but many cardiomyocytes express components necessary for SARS-CoV-2 uptake and replication. Although expression of the transmembrane serine protease TMPRSS2 is low in the heart, ACE2 and other auxiliary proteases (e.g., ADAM17, FURIN, NRP1, CTSL) known to be involved in viral activation, membrane fusion, and integrin coreceptors are highly expressed in cardiac tissue [9, 10, 11]. ADAM17 (a disintegrin and metalloprotease 17) was found to mediate the proteolysis and ectodomain shedding of ACE2; its activity was upregulated after SARS-CoV bound to ACE2 and promoted viral entry, whereas knockdown of ADAM17 by siRNA severely attenuated viral entry into cells [12, 13]. It is also noteworthy that in cells lacking TMPRSS2 expression, tissue proteinase L (CTSL) similarly promotes human coronavirus SARS-CoV and SARS-CoV-2 infection of host cells via a slow acid-activated pathway [14, 15, 16, 17]. However, the internal mechanism of SARS-CoV-2 invasion of myocardial tissue and how clinical features such as cardiovascular complications affect SARS-CoV-2 infection are not yet fully understood.

Individuals differ widely in the clinical consequences of infection, from asymptomatic illness to death. The severity and mortality of COVID-19 are closely related to CVD. A prospective, multicenter cohort study reported that 12.3% of 73,197 COVID-19 inpatients had cardiovascular comorbidities [18]. However, the prognosis of CVD in patients with COVID-19 seems to be controversial. Some studies have found that myocardial injury is significantly correlated with the fatal outcome of COVID-19, and patients with underlying CVD but no myocardial injury have a better prognosis [19]. Therefore, it is necessary to further explore the mechanism of SARS-CoV-2 infection of myocardial tissue to reveal the impact of cardiovascular diseases on COVID-19. Factors such as age and sex are also considered to be associated with the severity and mortality of COVID-19 [18]. We can better understand COVID-19 tropism and illness outcome heterogeneity by identifying the specific cell types that can be infected by SARS-CoV-2 and correlating proteins critical to SARS-CoV-2 infection with important variables such as age and sex [20].

Limited by the difficulty of obtaining human heart samples and the natural differences between other biological models and the human body, research on the molecular mechanisms of human heart disease has been hindered [21]. Utilizing single-cell RNA-sequencing (scRNA-seq) datasets from human cardiac samples may be one of the avenues to overcome the above problems. In this study, the first single-cell meta-analysis of the human heart and cell-type-specific expression patterns of ACE2 and auxiliary proteases were mapped by comprehensive analysis of the scRNA-seq datasets of 10 CVD patients.

## Results

### An overview of the cellular composition of the heart in healthy and CVD patients

**Fig. 1:**
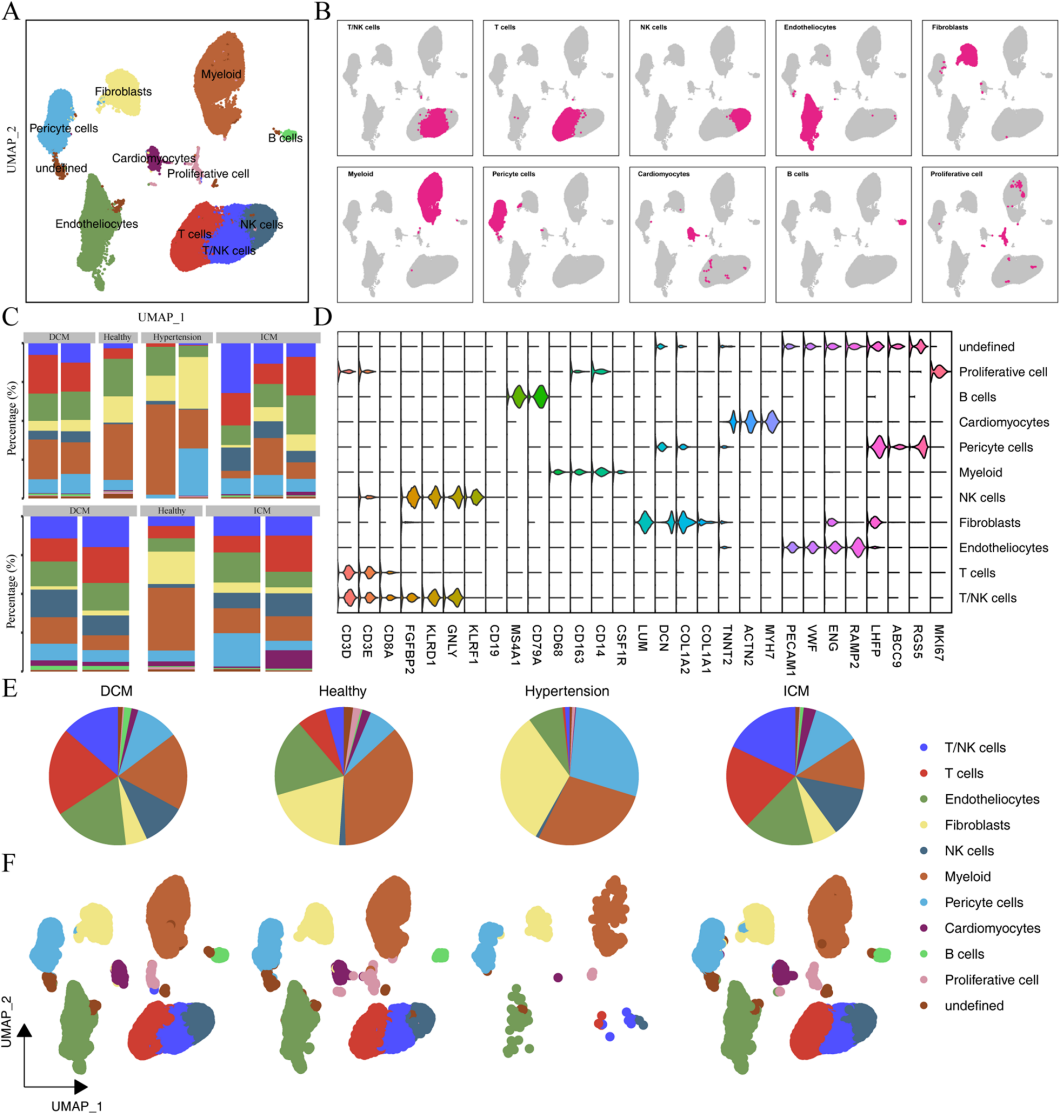
The landscape of the cellular composition of the heart from healthy and CVD patients at a single-cell resolution. **A **UMAP plot showing annotated cardiomyocyte types; **B** Distribution of each cell type of cardiomyocytes in different disease states; **C** Proportions of different cell types in the left ventricle (up) and right ventricle (down); **D** Violin plot generated from the comprehensive dataset showing the signature marker genes for each identified cell population; **E** Pie chart of the proportions of cardiomyocyte types in different disease states; **F** UMAP diagram of the proportions of cardiomyocyte types in different disease states

The two single-cell datasets GSE145154 and GSE134355 contain tissue samples from 9 patients, including 2 dilated cardiomyopathy (DCM) tissues, 2 ischemic cardiomyopathy (ICM) tissues, 2 hypertension (hypertension) tissues, 1 healthy heart tissue and 2 fetal normal heart tissues. After standard quality control, the software program harmony was used to remove the batch effect, and resolution = 1 was selected for dimensionality reduction clustering. The data were coaggregated into 25 subgroups. A total of 10 single-cell subpopulations were obtained after reviewing the literature for manual annotation (Fig. 1D), including cardiomyocytes, endothelial cells, pericytes, fibroblasts, myeloid cells, proliferating cells, T cells, B cells, and NK cells (Fig. 1A, B).

By comparing the cell components of the left and right ventricles, we found that the proportion of B cells, cardiomyocytes, and fibroblasts in the left and right ventricle tissues of DCM was relatively low, the proportion of myeloid cells and T cells in the left ventricle tissue was relatively high, and the right ventricular tissue had a similar proportion of remaining cells. In tissues with ICM, the proportion of B cells, cardiomyocytes, and fibroblasts in the left and right ventricle tissues was relatively low, and the proportion of T cells in the left ventricle was higher than that in the right ventricle. The proportions of cells in the left and right ventricle tissue in healthy people were similar, and only the proportion of endothelial cells in the left ventricle was higher than that in the right ventricle (Fig. 1C). Hypertensive tissues had a higher proportion of pericytes and fibroblasts than DCM, ICM, and healthy tissues. DCM and ICM had higher proportions of T cells than normal tissues (Fig. 1E, F).

### Distribution of double-positive ACE2^+^ -ADAM17^+^ and ACE2^+^ -CTSL^+^ cardiomyocytes associated with COVID-19

**Fig. 2:**
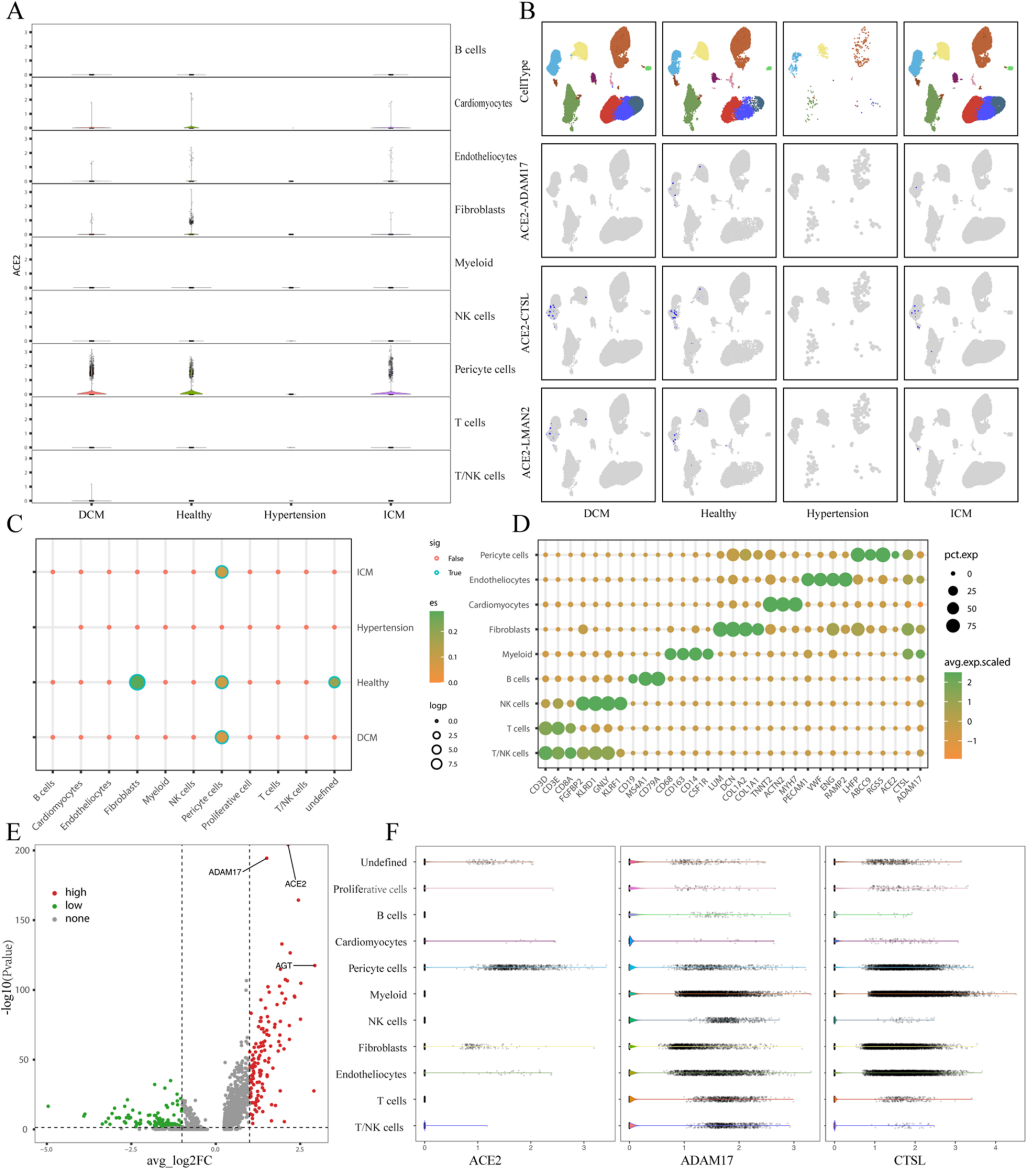
Distribution of ACE2, ADAM17 and CTSL. **A** Violin plot and scatterplot of ACE2 expression in different cell types in DCM, ICM, hypertension and healthy myocardial tissue; **B** *ACE2*^*+*^*ADAM17*^*+*^, *ACE2*^*+*^*CTSL*^*+*^ and *ACE2*^*+*^*-LMAN2*^*+*^ cells in different states and cell types, where blue represents double-positive cells; **C** Correlation between *ACE2*^*+*^*ADAM17*^*+*^ and cell type in DCM, ICM, hypertension and healthy myocardial tissue; **D** Marker genes of different cell types and the expression of ACE2, ADAM17 and CTSL in different cell types; **E** Volcano plot of the differential analysis of *ACE2*^*+*^*ADAM17*^*+*^ cells relative to all others; **F** Violin plot and scatter plot of ACE2, ADAM17 and CTSL expression in different cardiomyocyte subtypes

SARS-CoV-2 invasion of cells requires ACE2 and auxiliary proteases (ADAM17 and CTSL), whose expression may reveal viral thalassophilia and effects on cardiac tissue. To explore the distribution of *ACE2*^*+*^*-ADAM17*^*+*^ and *ACE2*^*+*^*-CTSL*^*+*^ in different cell types, we first drew the violin plot of ACE2 in different disease states and cell types (Fig. 2A). In myocardial tissue under different CVDs, ACE2 was expressed most highly in pericytes, but due to the small number of cells in hypertensive tissue, no significant changes could be seen. For the coexpression of *ACE2*^*+*^*-ADAM17*^*+*^ and *ACE2*^*+*^*-CTSL*^*+*^, we first counted the double-positive cells in different CVD tissue types and then visualized the distribution status of double-positive cells using UMAP plots (Fig. 2B). The results showed that *ACE2*^*+*^*-ADAM17*^*+*^ and *ACE2*^+^*-CTSL*^+^ cells were also mostly distributed in pericytes.

We also analyzed the coexpression of *ACE2* and *ADAM17*^*+*^ in different cell types under different disease states (Fig. 2C). The results revealed strong coexpression levels of *ACE2*^*+*^*-ADAM17*^*+*^ in pericytes in DCM, ICM and healthy tissues. The same result was also found in the dot plots of the marker genes of different cell types, and the proportion of *ACE2*^*+*^*ADAM17*^*+*^ and *ACE2*^*+*^*CTSL*^*+*^ in pericytes were relatively high (Fig. 2D). The violin plots of ACE2, ADAM17 and CTSL in different cell types also showed that all three genes were expressed in pericytes (Fig. 2F).

To clarify the genes specifically expressed in *ACE2*^*+*^*-ADAM17*^*+*^ cells relative to other cells (Additional file 1: Table S1), we performed differential analysis using the FindMarkers function in Seurat v4.0. This identified a total of 246 differential genes with |logFC| > 1 and adj. P < 0.05 as the screening criteria, of which 132 were upregulated and 114 were downregulated. Among them, both ACE2 and ADAM17 showed the most significant differences among the upregulated genes (Fig. 2E). This outcome suggested that in myocardial tissue, pericytes may be specific target cells for SARS-CoV-2 invasion of cardiac tissue.

### Correlation of ACE2, ADAM17 and CTSL expression in cardiomyocytes with age, sex and cardiovascular comorbidities

**Fig. 3:**
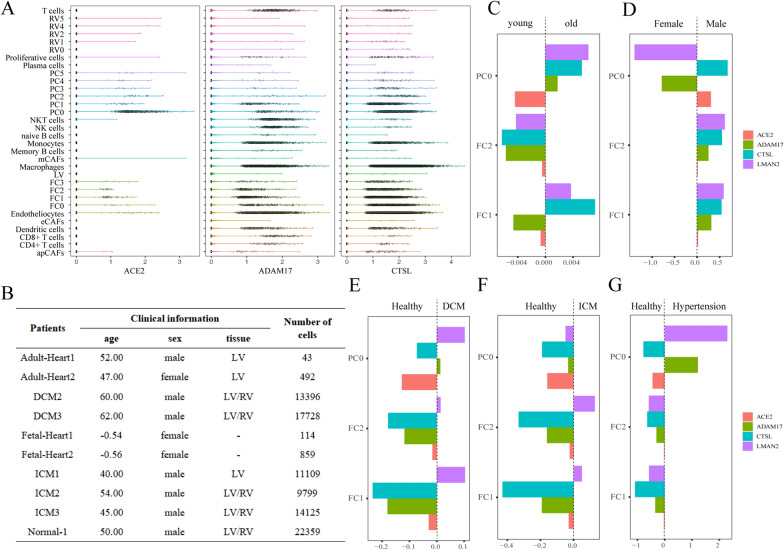
Correlations of ACE2, ADAM17 and CTSL with clinical characteristics. **A** Violin and scatter plots of ACE2, ADAM17 and CTSL expression in 32 cardiomyocyte subtypes; **B** Clinical information for all patients whose single-cell data are used; **C**, **D** Correlations of age, sex, and different cardiovascular comorbidities with gene expression of ACE2 (orange), ADAM17 (green), CTSL (cyan) and LMAN (purple) in 3 cardiomyocyte subtypes

Next, we explored how the expression of ACE2, ADAM17 and CTSL in specific cell subsets correlated with three key clinical features (age, sex, and cardiovascular comorbidities) that might make COVID-19 more severe. First, we reclustered all cell types using harmony’s method for descending clustering and subdivided them according to resolution, which resulted in a total of 32 cell subtypes. Then, we reanalyzed the distribution of ACE2, ADAM17 and CTSL in the 32 cell subtypes (Fig. 3A). We found higher expression of ACE2, ADAM17 and CTSL in three cell subtypes, PC0 (Pericyte cells), FC1 (Fibroblast cells) and FC2. Therefore, in these three cell subtypes, we calculated the correlations of ACE2, ADAM17, and CTSL gene expression with clinical characteristics using a mixed-effects model. The clinical information of each single-cell sample is shown in Fig. 3B. Examining the association trend of ACE2 and auxiliary protease genes (ADAM17, CTSL, and LMAN2), ACE2 expression and age in the three pericyte subtypes showed no obvious trend (Fig. 3C). ACE2 and CTSL coexpression showed a clear trend of joint upregulation in males (Fig. 3D). ACE2, ADAM17, and CTSL all showed a clear trend of joint upregulation in healthy tissues compared with DCM, ICM, and hypertension (Fig. 3E–G). These results suggested that among the clinical features of COVID-19, the expression trend of ACE2 and auxiliary proteases was more obvious in male patients. Age and cardiovascular comorbidities did not increase the expression trend of ACE2 or auxiliary proteases.

### Cell-type-specific expression of extra proteases that may be relevant to infection

**Fig. 4:**
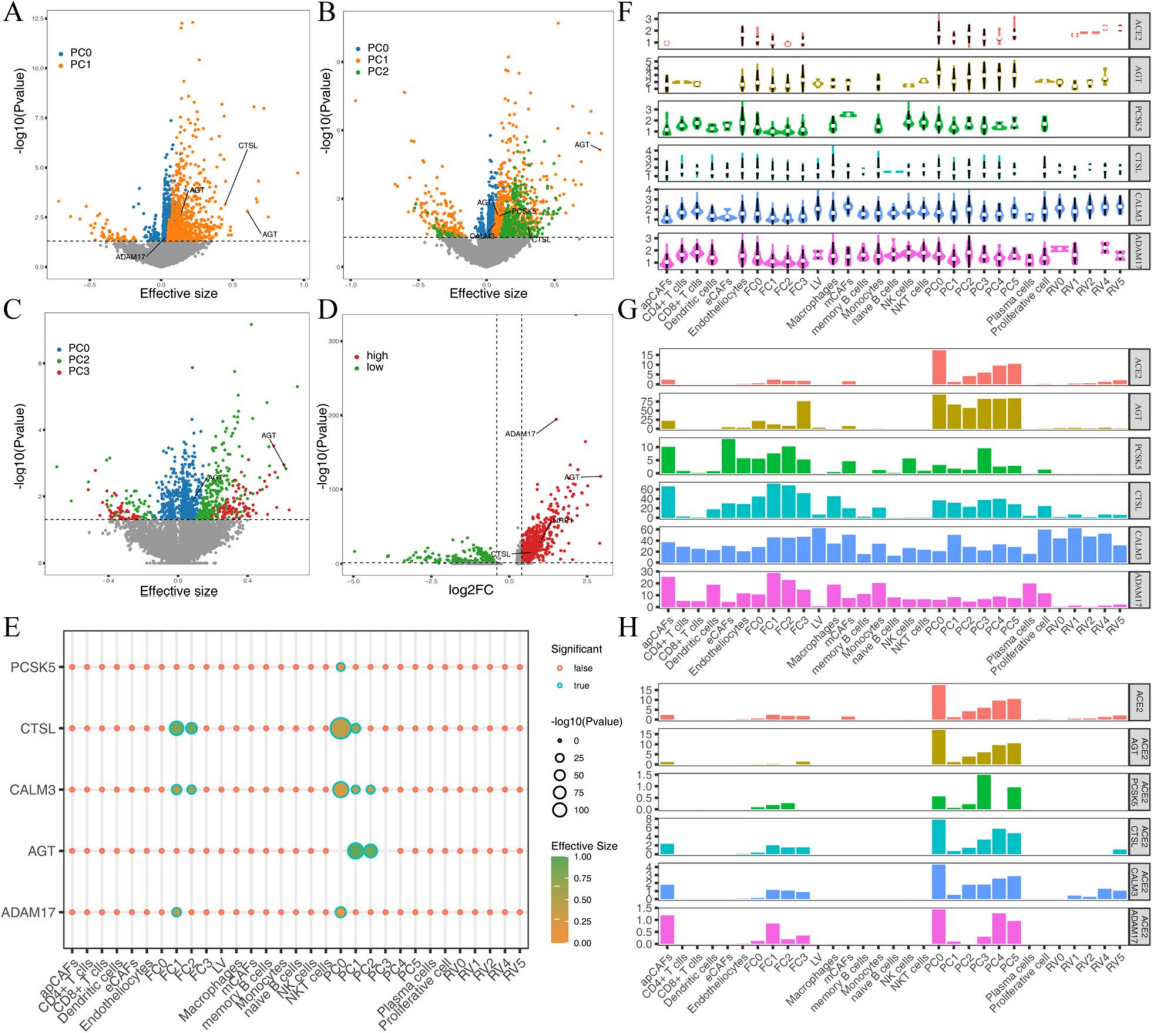
Cell type-specific expression of other proteases. **A**–**C** Heat map of ACE2-related genes in ICM, DCM, and pericyte subsets of healthy tissues; **D** Heat map of differential gene expression analysis between *ACE2*^*+*^*ADAM17*^*+*^ cells and other cardiomyocytes; **E** Correlation analysis between ACE2 and other gene expression in different cardiomyocyte types; **F** Violin plot of expression levels of ACE2, AGT, PCSK5, CTSL, CALM3 and ADAM17 genes; **G** Histogram of cell proportions expressing the ACE2, AGT, PCSK5, CTSL, CALM3 and ADAM17 genes; **H** Histogram of the proportions of double-positive cells

Infection by SARS-CoV-2 of myocardial tissue may involve complex mechanisms, and additional proteases likely play roles in proteolytic cleavage of viral proteins for entry and egress [7, 22]. To search for such proteases, we constructed random effects models and plotted heat maps for ACE2 and all eligible genes in DCM, ICM, and healthy human pericytes. In the PC0 pericyte subtype of the ICM, ACE2 expression correlated with the expression of ADAM17 and AGT, and in the PC1 pericyte subtype, ACE2 expression correlated with the expression of AGT and CTSL. In the PC0 pericyte subtype of DCM, ACE2 expression correlated with the expression of AGT and CALM3. In the PC1 pericyte subtype, ACE2 expression correlated with the expression of PCSK5, AGT and CTSL. In the PC2 pericyte subtype, there were no expressed genes associated with ACE2. ACE2 expression correlated with AGT expression in the PC0 and PC2 pericyte subtypes of healthy tissues (Fig. 4A–C). Because of the low cell count in hypertensive patients, there were no eligible pericytes from them.

According to the differential gene expression analysis between *ACE2*^*+*^*-ADAM17*^*+*^ cells and other cells, AGT, CTSL and NRP1 were also highly expressed in *ACE2*^*+*^*-ADAM17*^*+*^ cells (Fig. 4D). We also analyzed the correlation between ACE2 and other gene expression in all cell types (Fig. 4E). ACE2 expression was positively associated with the number of cells with both CTSL and CALM3 gene expression, and the effect values associated with ACE2 expression and CTSL expression were the highest in the PC0 pericyte subtype.

To visualize the expression levels of related protein genes in different cell types, we plotted the expression levels of different genes (ACE2, AGT, PCSK5, CTSL, CALM3 and ADAM17) in the 32 cardiomyocyte subtypes (Fig. 4F), the proportion of cells expressing related genes (Fig. 4G) and the proportion of double-positive cells coexpressing ACE2 (Fig. 4H). ACE2 and AGT were mainly expressed in pericytes and fibroblasts, while PCSK5 was not expressed in B cells, plasma cells, or cardiomyocytes. CTSL, CALM3, and ADAM17 were expressed to varying degrees in all cell types. The results of the cell occupancy plot of the expressed genes were similar to the gene expression levels.

### Enrichment analysis

**Fig. 5:**
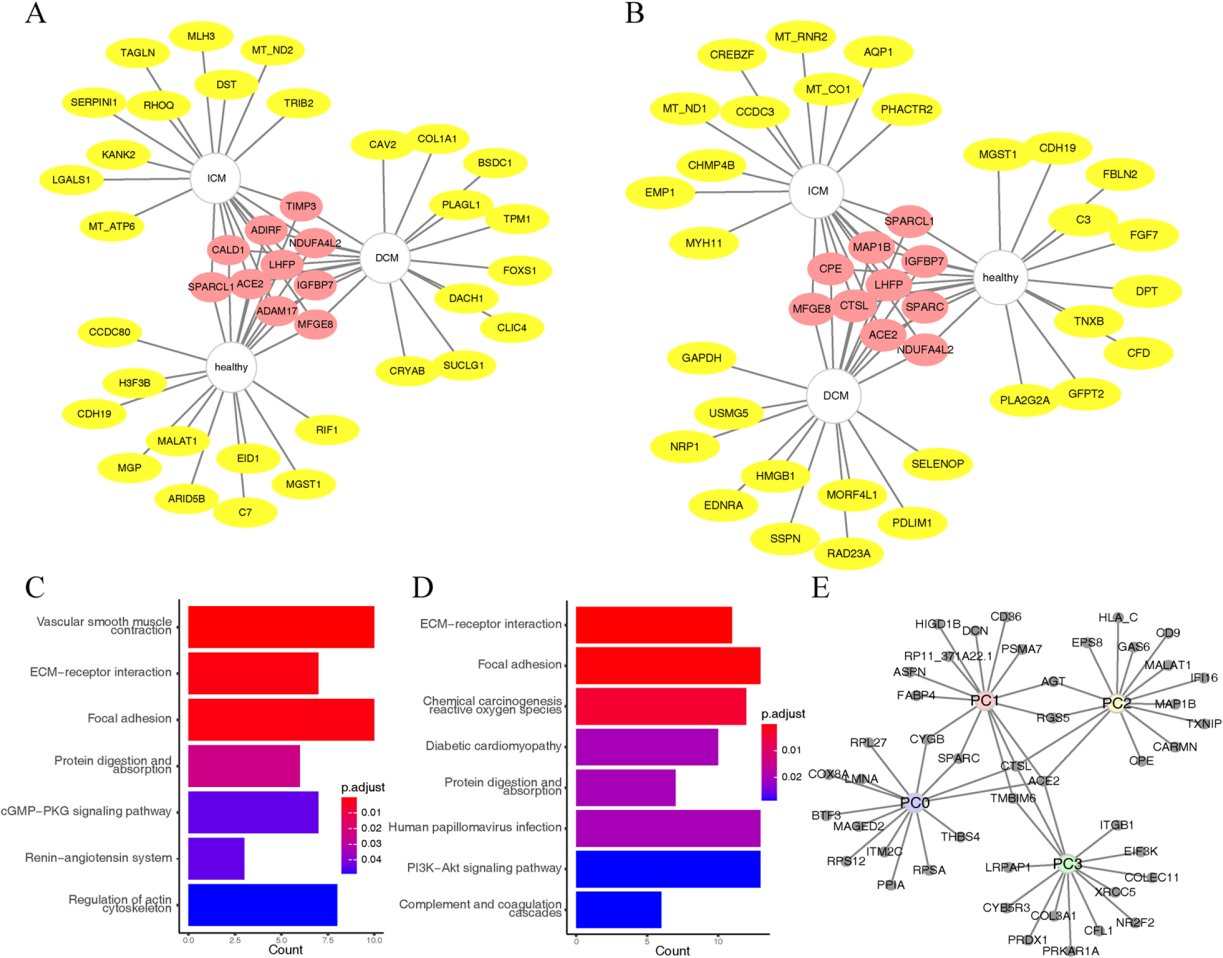
Enrichment analysis of ACE2^***+***^ADAM17^+^ and ACE2^+^CTSL^+^ cells. **A** *ACE2*^*+*^*ADAM17*^*+*^ cell-related genes in different tissues; **B** *ACE2*^*+*^*CTSL*^*+*^ cells in different tissues with associated genes; **C** *ACE2*^*+*^*ADAM17*^*+*^ cell-related gene KEGG enrichment results; **D** *ACE2*^*+*^*CTSL*^*+*^ cell-related gene KEGG enrichment results; **E** Genes associated with *ACE2*^*+*^*CTSL*^*+*^ cells in different cardiomyocyte subtypes

We used a random forest modeling approach for functional enrichment of *ACE2*^*+*^*-ADAM17*^*+*^ cells and *ACE2*^*+*^*-CTSL*^*+*^ cells (Fig. 5A–D). In DCM, ICM and healthy tissues, there were both commonalities and particularities of genes associated with *ACE2*^*+*^*-ADAM17*^*+*^ and *ACE2*^*+*^*-CTSL*^*+*^ cells. We input their common genes for KEGG functional enrichment. *ACE2*^*+*^*-ADAM17*^*+*^ cells were mainly enriched in ECM-receptor interaction, focal adhesion and vascular smooth muscle contraction. *ACE2*^*+*^*-CTSL*^*+*^ cells were mainly enriched in oxidative stress (chemical carcinogenesis reactive oxygen species), inflammatory response (PI3K-Akt signaling pathway) and coagulation-related pathways (complement and coagulation cascades). Then, we analyzed *ACE2*^*+*^*-CTSL*^*+*^ cell-associated genes in different pericyte subtypes using a mixed-effects model (Fig. 5E). These results suggest that the pathways of action of SARS-CoV-2 in myocardial tissue infection may focus on the pathways of inflammatory response, oxidative stress, intercellular matrix bonding, and vasoconstriction.

### Pericyte correlation analysis

**Fig. 6:**
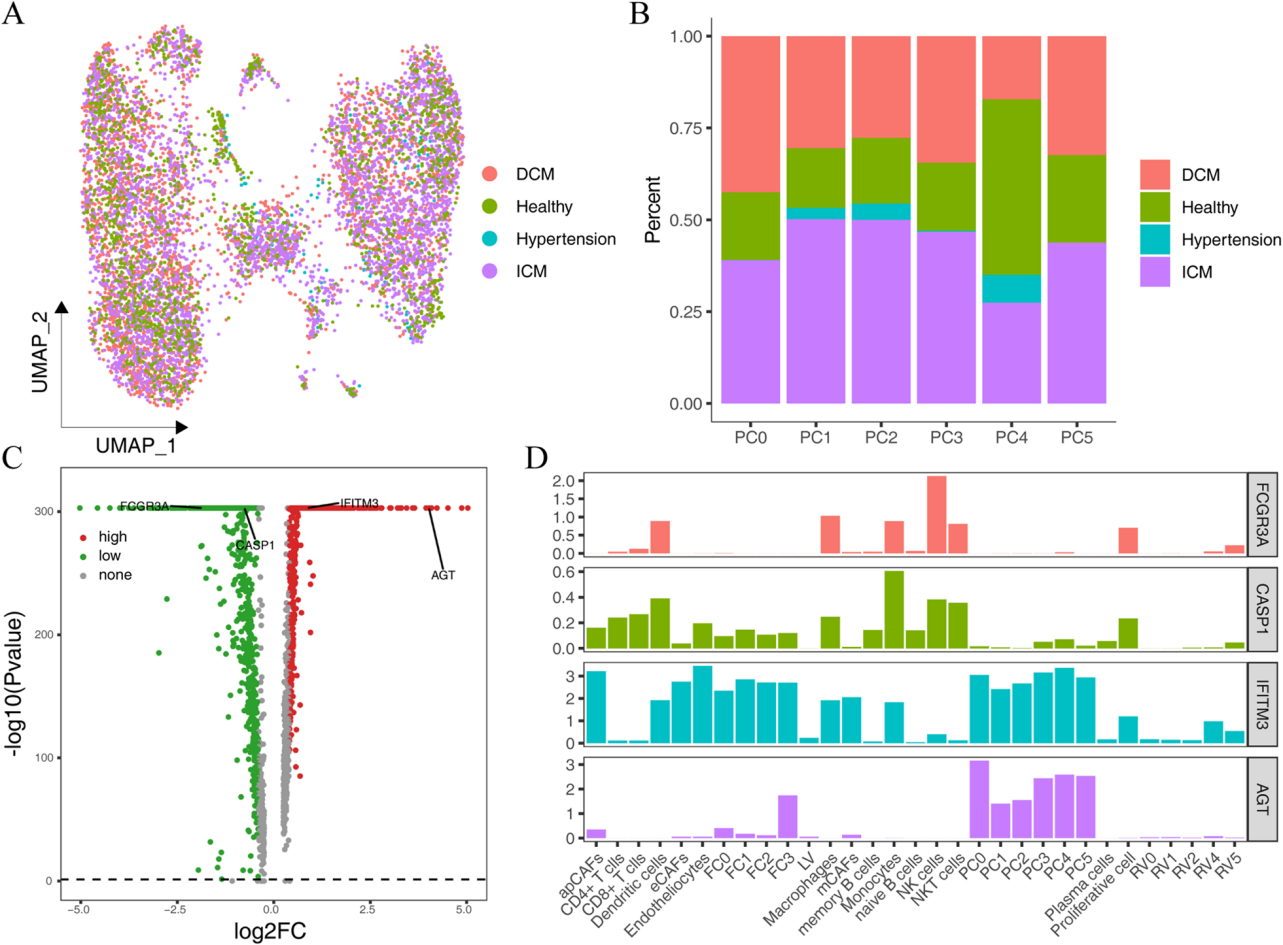
Subdivision of pericytes and expression of specific helicases. **A** Pericellular distribution after redimensionalization and clustering; **B** The proportion of pericyte subsets in different CVD states; **C** Heatmap of differential genes in pericytes relative to other cells; **D** The proportion of cells expressing differentially expressed helicases in different cell types

From the above results, we see that ACE2 and its related auxiliary proteases were mainly expressed in pericytes. Therefore, we redimensionalized the clustering of pericytes in the combined single-cell data and found that pericytes were more evenly distributed in DCM, ICM, hypertension and healthy tissues (Fig. 6A). Then, we compared the proportions of different subtypes of pericytes between the states and found that the proportion of the PC4 cell subpopulation in healthy tissues was higher than that in CVD tissues (Fig. 6B). To further analyze the helicases specifically expressed in pericytes, we compared the differentially expressed genes between pericytes and all other cells by using |logFC| > 0.4 and adj. P < 0.05 as the screening criteria. Only IFITM3 and AGT were highly expressed in pericytes, while CASP1 and FCGR3A had low expression in pericytes (Fig. 6C), which was also supported by histograms of the proportion of cells expressing these genes in different cell types (Fig. 6D).

### Cell–cell communication

**Fig. 7:**
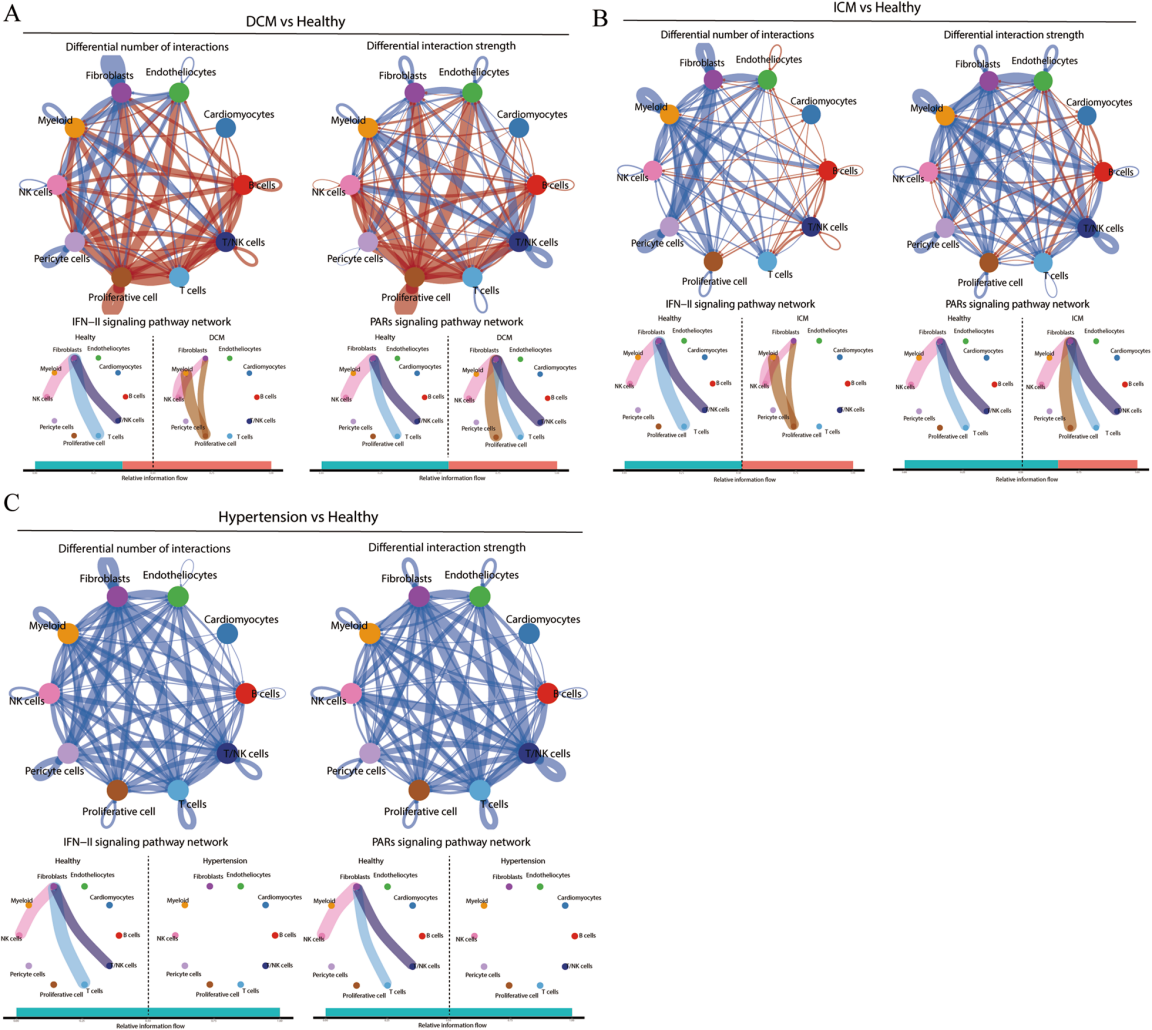
Differences in cell‒cell communication between DCM, ICM, hypertension and healthy tissue. **A**–**C** DCM, ICM and hypertension compared with healthy tissue, the intensity and number of cell‒cell communication differences between different cell types, and the differences in the IFN-II signaling pathway and PAR signaling pathway

We further analyzed the cell‒cell communication of different CVD states relative to healthy tissues. Compared with healthy tissues, the communication between pericytes and B cells is enhanced in DCM, while the communication between fibroblasts was weakened. In addition, the IFN-II signaling pathway was enriched in DCM tissue, while the PAR signaling pathway was not significantly different between healthy tissue and DCM (Fig. 7A). Communication between pericytes and fibroblasts was significantly weaker in ICM relative to healthy tissues. The IFN-II signaling pathway was not significantly different between healthy tissues and ICM, whereas the PAR signaling pathway was weaker in ICM than in normal tissues (Fig. 7B). Communication between both pericytes and other cells was diminished in hypertensive tissues relative to healthy tissues, and the IFN-II signaling pathway and PAR signaling pathway were enriched to a much higher extent in healthy tissues than in hypertensive tissues (Fig. 7C). Analysis of pericyte and intercellular communication suggested that SARS-CoV-2 reaches myocardial tissue and affects pericytes precisely due to IFITM3- and AGT-related pathways and that different clinical outcomes arise between different cardiovascular comorbidities due to differences in IFN-II and PAR signaling in fibroblasts.

## Discussion

This study reveals, for the first time at single-cell resolution, the factors that affect the entry of SARS-CoV-2 into cardiac tissue cells in different CVD states. Single-cell technologies offer powerful new tools to dissect cell types that reside within healthy and diseased tissues [23, 24]. In recent years, this approach has been leveraged to provide a deeper understanding of the cellular composition of the healthy human heart, but deciphering how cardiovascular disease affects the cardiac cellular transcriptional landscape has been hampered by limited sample data [25, 26]. By analyzing approximately 90,000 single cells from different cardiac regions of 10 donors with DCM, ICM, hypertension, and healthy states, we identified 10 major cardiomyocyte types and revealed their cell type-specific transcriptional programs. Because of the low expression of TMPRSS2 in myocardial tissue, we used coexpressed forms of ACE2 and auxiliary proteases (ADAM17 and CTSL) to locate specific cell types—pericytes and fibroblasts—that SARS-CoV-2 might invade. Based on specific cell types (PC0, FC1 and FC2), we correlated key factors of SARS-CoV-2 invasion of host cells (ACE2, ADAM17 and CTSL) with key Thank you for your valuable feedback on the Introduction section of the manuscript.covariates such as age, sex and cardiovascular comorbidity and found the correlations between them. In addition, the expression of other potential auxiliary proteases may help in the search for therapeutic possibilities related to the disruption of viral processing by protease inhibition. This study identified AGT, CALM3, PCSK5, NRP1 and LMAN as proteases coexpressed with ACE2. The enrichment analysis revealed that relevant immune pathways involved in viral infection might include the extracellular matrix interaction pathway, adherent plaque pathway, vascular smooth muscle contraction inflammatory response and oxidative stress. Correlation analysis of pericytes revealed specific high expression of IFITM3 and AGT in pericytes. Finally, cell‒cell communication experiments revealed the presence of IFN-II signaling pathway and PAR signaling pathway differences in fibroblasts from different cardiovascular comorbidities.

Pericytes are mural cells that cover and adhere to the basement membrane of the cardiac microcirculation (including terminal microarteries, precapillary microvenules and capillaries) and have the function of regulating blood flow and vascular permeability [27]. Pericytes may be one of the first cells contacted by virus particles entering myocardial tissue through the blood circulation. Due to the high expression of ACE2, ADAM17, CTSL and related auxiliary proteases, SARS-CoV-2 infects and replicates in pericytes. In an autopsy report of a patient with COVID-19, it was found that SARS-CoV-2 was located in the interstitial cells of myocardial tissue rather than cardiomyocytes in slices of cardiac tissue [28]. Despite the antiviral function of the pericyte-specific, highly expressed IFITM3 protein, the human IFITM3 protein exhibits a pro-viral effect, i.e., enhanced viral fusion at the plasma membrane [29, 30]. Endocytosis of IFITM3 promotes mutation of residues in its YxxФ motif, converting human IFITM3 into an enhancer of SARS-CoV-2 infection, and cell-to-cell fusion assays confirm that endocytic mutants enhance spike-mediated fusion with the plasma membrane [30]. The genetic variant rs12252-C of IFITM3 is associated with more severe COVID-19, possibly by causing defects in the control of viral replication in cells [31]. Angiotensinogen, expressed by AGT, is an important component of the RAAS and is a potent regulator of blood pressure, fluid balance, and electrolyte homeostasis. The specific high expression of AGT and the interaction between pericytes and endothelial cells may be one of the reasons why SARS-CoV-2 infection of pericytes leads to vasoconstriction and decreased myocardial blood flow [27]. In addition, it has been shown that the S-glycoprotein of SARS-CoV-2 induces pericardial cells to produce proinflammatory cytokines, including MCP1, IL-6, IL-1β, and TNF-α, which are important components of the cytokine storm associated with respiratory failure and high mortality in patients with COVID-19 [32].

This study used a mixed-effects model to attempt to correlate key factors (ACE2, ADAM17, and CTSL) for SARS-CoV-2 invasion of host cells with key covariates such as age, sex, and cardiovascular comorbidities. Sex is an important factor affecting SARS-CoV-2-infected cells. In the present study, the expression of ACE2 and auxiliary proteases clearly trended higher in male patients. Epidemiological studies have found that men are more likely to be infected than women, and they account for the majority of severe illnesses and deaths [33]. Surprisingly, the expression of ACE2 and auxiliary proteases in healthy myocardial tissue in this study showed a more pronounced trend for various cardiovascular comorbidities. Previous studies have focused more on myocardial damage by COVID-19, ignoring the effect of cardiovascular comorbidities themselves on the virus. Mortality from COVID-19 among adults with congenital heart disease (CHD) is commensurate with that in the general population [34]. A possible explanation for this phenomenon is that preconditioning of the underlying CVD paradoxically protects the myocardium from damage caused by SARS-CoV-2, thereby mitigating the overall impact on the myocardium [34, 35]. Historical data from previous viral epidemics confirm adverse pulmonary and cardiovascular effects in CVD patients [36], but this phenomenon may be due to an overwhelming immune-inflammatory cytokine response, direct viral invasion of cardiomyocytes, or poor myocardial oxygenation from severe hypoxia due to lung injury. These mechanisms might plausibly complicate patients with CHD who were already prone to myocardial dysfunction, limited myocardial oxygenation, or pulmonary vascular disease. We attempted to reduce the background variance and unbalanced distribution of explanatory covariates for the proposed model. Potential confounders and limited sample size made the modeling of clinical characteristics factors crude. A larger dataset may help get rid of the above issues. Therefore, there is a need to further design reasonable experiments to validate the actual effect of cardiovascular comorbidities on COVID-19.

Finally, the expression of other potential auxiliary proteases may contribute to the generation of therapeutic hypotheses related to the disruption of viral processing through protease inhibition. The S glycoprotein of SARS-CoV-2 contains two subunits, S1 and S2, and NRP1 can bind to the furin cleavage site of the S1 subunit, which can promote SARS-CoV-2 infection [7]. Because PCSK5 proteases are located in different membrane compartments, they may process the S glycoprotein of SARS-CoV-2 at different viral stages [37]. The protein CALM3, which is significantly coexpressed with ACE2, is part of the calcium signal transduction pathway, which mediates the control of a large number of enzymes, ion channels, aquaporins and other proteins through calcium binding [38]. LMAN can participate in biological processes such as protein metabolism, Golgi transport dynamics and subsequent protein modification. In addition, fibroblasts are among the specific cells that SARS-CoV-2 may first invade by virtue of their double-positive protein localization [23, 28]. We observed significant differences in the IFN-II signaling pathway and PAR signaling pathway in fibroblasts under different cardiovascular mergers by cell‒cell communication analysis. One of the hallmarks of severe COVID-19 is a persistent interferon (IFN) response [39]. An innate error affecting human fibroblast IFN immunity may underlie life-threatening COVID-19 pneumonia in patients without prior severe infection [40]. Protease-activated receptors (PARs) may enhance platelet activation through thrombin-mediated platelet calcium mobilization in injured myocardium [41].

This study still has some limitations that should be mentioned. First, it attempted to reveal the influence of underlying cardiovascular disease factors alone on SARS-CoV-2 infection of myocardial tissue. SARS-CoV-2-infected tissues were not used. SARS-CoV-2 that has infected other tissues may instruct the cell to start replicating its genome, produce its proteins and assemble them into many new copies of the virus, which, upon release, can circulate in the blood and affect myocardial tissue after release [32]. Therefore, we tried to rule out the influence of SARS-CoV-2-infected tissue data. Second, the difficulty of obtaining human heart samples restricted the sample size of this study. However, studies like this may be one of the ways to solve the limitation of sample size: by referring to previous studies, using published research data and using the research model of coexpression of virus and key proteins [42]. A strength of this study is that it used an innovative research model of the mechanism by which SARS-CoV-2 affects myocardial tissue, and it excluded some negative factors of the impact of SARS-CoV-2 infection on myocardial tissue.

In conclusion, this study is the first to correlate cell type-specific changes in expression levels with age, sex, and cardiovascular disease. It provides new insight into the pathway by which SARS-CoV-2 infects myocardial tissue. Our meta-analysis provides a detailed molecular and cellular map to help us better understand the invasion, pathogenesis, and association with clinical features of SARS-CoV-2 in myocardial tissue.

## Methods

### Published dataset and patient samples

Sample collection was reviewed and approved by the Institutional Review Board (IRB) at the institution where the sample was originally collected. GSE145154 was approved by the Ethics Committee of Fuwai Hospital in Beijing, China. Tissue samples of hearts with DCM and ICM in the GSE145154 dataset were obtained from patients undergoing transplant, while these causes of DCM excluded patients: cardiac amyloidosis, cardiac sarcoidosis, viral myocarditis, giant cell myocarditis, peripartum cardiomyopathy, chemotherapy-associated cardiomyopathy, obesity, diabetic cardiomyopathy, arterial coronary disease, valvular disease, and congenital heart disease. GSE134355 was approved by the Research Ethics Committee of the Zhejiang University School of Medicine, Research Ethics Committee of the First Affiliated Hospital, Research Ethics Committee of the Second Affiliated Hospital and Research Ethics Committee of Women’s Hospital at Zhejiang University (Approval Number: 20,170,029, 2,01,80,017, 2,01,90,034, 2,01,80,15, 2,01,85,07, 2,01,87,66 and 2,01,81,85). Informed consent for fetal tissue collection and research was obtained from each patient after her decision to legally terminate her pregnancy but before the abortive procedure was performed. Informed consent for collection and research of surgically removed adult tissues was obtained from each patient before the operation. Informed consent for the collection and research of tissues from deceased-organ donation was obtained from the donor family after the cardiac death of the donor.

Publicly available single-cell RNA-seq datasets were downloaded from the Gene Expression Omnibus (GEO) [43]. GSE145154 was sequenced on the Illumina HiSeq 6000 and HiSeq X Ten platforms using 10x Genomics technology, including 2 DCM tissues, 2 ICM tissues, and 1 healthy heart tissue, with left and right ventricular samples taken from each patient for sequencing. GSE134355 was sequenced on the HiSeq X Ten platform using 10x Genomics technology, including 2 adult hypertensive patient heart tissue and 2 fetal normal heart tissue.

### Integrated analysis of published datasets

The single-cell data used were the original UMI count data. Its preprocessing, quality control, normalization, and dimensionality reduction clustering were all performed using the Scanpy package (v4.0) [44]. The quality control standards were as follows: (1) Each gene must be expressed in at least 3 cells. (2) At least 500 genes were expressed in each cell. (3) The variable nfeatures and the counts of each sample were according to median ± 3*MAD (median absolute deviation) standard screening. (4) The mitochondrial gene proportion was 10% as a threshold. (5) The hemoglobin gene proportion was 1% as a threshold. The subsequent data standardization, normalization, search for hypervariable genes, and dimensionality reduction clustering were all done according to the default parameters and standard procedures of the Seurat package.

The log1p function ln(10,000 × g_ij_ + 1) and column sum were used to log-normalize (UMIs/10,000 + 1) each dataset, where a gene’s expression profile g is the outcome of the UMI count for each gene i, for cell j, normalized by the total of all UMI counts for cell j. We use the harmony-pytorch Python implementation (v0.1.1; https://github.com/lilab-bcb/harmony-pytorch/) of the Harmony scRNA-seq integration method for batch correction to integrate data between different samples, and selected the first 30 principal components and resolution = 1 for dimensionality reduction clustering [45]. Single-cell group naming was done by reading papers to collect marker genes and manually annotating them.

### Differential gene expression analysis

To further analyze the differentially expressed genes among cell populations, we used the FindMarkers function in Seurat v4.0 for analysis. The selection criteria for differential genes were adj. p < 0.05, and the selection criteria for logFC were based on an earlier report [44].

### Coexpression analysis across diseases and cell types

We collected single-cell sequencing data of 3 CVDs and normal cardiomyocytes. To evaluate the coexpression of ACE2 and ADAM17 in different cell types and different disease conditions, we selected cell types with more than 15 ACE2^+^ cells for analysis, and ACE2- cells were selected by downsampling according to clinical characteristics using the ROSE package. We employed a mixed model with a random intercept that differed for each donor to account for donor-specific effects (i.e., batch effects):

$${\text{Y}}_{\text{i}} \sim \text{A}\text{C}\text{E}2 + \left(1\right|\text{S})$$

Where ACE2 represents the binary coexpression state of each cell (that is, double-positive versus double-negative cells), Yi represents the expression level of gene i in cells, expressed in units of log2(transcripts per 10,000 reads (TP10K) + 1), and S represents the donor from which each cell was isolated. The specific implementation used the lme4 package of R software for analysis [46].

### Integrated analysis for associating ACE2, ADAM17 and CTSL expression with age, sex and cardiovascular comorbidities

We combined all scRNA-seq datasets of human left and right ventricular cells, as well as fetal samples, including the expression counts of just the above three genes, to analyze the relationships between age, sex, and cardiovascular comorbidities and the expression of ACE2, ADAM17, and CTSL. First, to refine the localization of *ACE2*^*+*^*ADAM17*^*+*^ cells, we subdivided each cell subpopulation of single cells, integrated data between different samples of the same cell type using the harmony package, and selected the top 30 main adult components and the unique resolution of each cell population for dimensionality reduction clustering [45]. Single-cell group naming was done by reading papers to collect marker genes and manually annotating them. Then, the expression levels of ACE2, ADAM17, and CTSL in different cell subpopulations were plotted according to the results of subgroup segmentation, and the subpopulation with a higher content of double-positive cells was selected to explore the relationship between *ACE2*^*+*^*ADAM17*^*+*^ cells and clinical characteristics. The data imbalance was also treated using the downsampling method in the ROSE package, and the relationship between the two was assessed using the mixed-effects model in the lme4 package [46].

$${\text{Y}}_{\text{i}} \sim {\text{X}}_{\text{i}} + \left(1\right|\text{d}\text{o}\text{n}\text{o}\text{r})$$

where Yi represents the expression level of the dichotomized genes, while Xi represents the different clinical features. The specific method can be found in the published literature [20].

### Coexpression of ACE2 and other auxiliary protease classes

Additional proteases may play a role in the proteolytic cleavage of viral protein entry and exit. To predict such proteases, we tested the coexpression of ACE2 with each of 625 annotated human protease genes [47]. To further analyze the coexpression of ACE2 and other protease classes, we assessed the coexpression of all genes and ACE2 in three disease tissue types and in healthy tissues using a random-effects model with the lme4 package [46]. The relationships between ACE2 and the PCSK family, CTSL, ADAM17, NRP1, HMGB1, CALM1, CALM3, KNG1, AAMP, NTS, AGT, DEFA5, SLC6A19 and other proteases were investigated. The relationships between ACE2 and these proteases in different cell types were also analyzed.

### Functional enrichment analysis of double-positive cells

To further analyze the functional enrichment in the double-positive cells, we selected *ACE2*^*+*^*-ADAM17*^*+*^ cells and *ACE2-ADAM17* cells in different tissues for differential analysis to obtain the differential genes. The numbers of *ACE2*^*+*^*-ADAM17*^*+*^ cells and *ACE2-ADAM17* cells were balanced by a downsampling method and then modeled using a random forest algorithm. The top 500 genes associated with double-positivity of cells were filtered by importance, and the intersection of the top 500 genes in different tissues and the genes specific to each tissue were calculated separately. Then, the top 10 genes ranked by the sum of importance were taken for visualization using Cytoscape software [48]. In addition, to further analyze the functional enrichment of double-positive cells, we input the common genes among the top 500 genes in different tissues for KEGG enrichment analysis, using the package clusterProfiler in R software [49]. The functional enrichment map of *ACE2*^*+*^*-CTSL*^*+*^ cells was drawn in the same way.

To identify the genes related to double-positive cells in different cell types, we found the genes related to double-positive cells in different cell types using the random effect model, sorted them according to the size of the effect value, and selected the first 12 genes using Cytoscape software [48].

### Analysis of cell‒cell communications

CellChat objects were created based on the pericyte UMI count matrix of each group (DCM, ICM, hypertension, and healthy) via CellChat (https://github.com/sqjin/CellChat, R package, v.1). The difference between the cell interaction of different diseased myocardial tissues and the cell interaction of normal myocardial tissue was calculated by the CellChat package. With “CellChatDB.human” set up as the ligand—receptor interaction database, cell‒cell communication analysis was then performed via the default settings. The total number of interactions was compared against interaction strength by merging the CellChat objects of each group by the function mergeCellChat. The visualization of the differential number of interactions or interaction strength among different cell populations was achieved by the function netVisual_diffInteraction. Finally, differentially expressed signaling pathways were found by the function rankNet, and the signaling gene expression distribution between different datasets was visualized by the function plotGeneExpression [50].

### Statistical analysis

All data calculations and statistical analyses in this study were done using R software (https://www.r-projec t.org/, version 4.1.2). All statistical P value values were two-sided, where differential genetic screening was considered statistically significant with a corrected P value < 0.05, and the P value standard values for the remaining statistical tests were as described in the text.

## Supplementary Information

**Additional file 1:** Raw Data. This file contains the raw data collected during the course of our research, which was used in the analysis presented in the paper.

## Data Availability

The original contributions presented in the study are included in the article/supplementary material. Further inquiries can be directed to the corresponding author.

## Abbreviations

ACE2: Angiotensin-converting enzyme 2
CHD: Congenital heart disease
COVID-19: Coronavirus disease 2019
CTSL: Proteinase L
CVD: Cardiovascular disease
DCM: Dilated cardiomyopathy
GEO: Gene Expression Omnibus
ICM: Ischemic cardiomyopathy
IFN: Interferon
IRB: Institutional Review Board
PARs: Protease-activated receptors
RAAS: Renin-angiotensin-aldosterone system
scRNA-seq: Single-cell RNA sequencing
